# Identification and Quantification of Extracellular Vesicles: Comparison of SDS-PAGE Analysis and Biosensor Analysis with QCM and IDT Chips

**DOI:** 10.3390/bios14080366

**Published:** 2024-07-27

**Authors:** Yaw-Jen Chang, Wen-Tung Yang, Cheng-Hsuan Lei

**Affiliations:** Department of Mechanical Engineering, Chung Yuan Christian University, Chung Li District, Taoyuan City 320314, Taiwan

**Keywords:** extracellular vesicles (EVs), SDS-PAGE, quartz crystal microbalance (QCM), interdigitated electrode (IDT)

## Abstract

This study presents and compares two methods for identifying the types of extracellular vesicles (EVs) from different cell lines. Through SDS-PAGE analysis, we discovered that the ratio of CD63 to CD81 in different EVs is consistent and distinct, making it a reliable characteristic for recognizing EVs secreted by cancer cells. However, the electrophoresis and imaging processes may introduce errors in the concentration values, especially at lower concentrations, rendering this method potentially less effective. An alternative approach involves the use of quartz crystal microbalance (QCM) and electroanalytical interdigitated electrode (IDT) biosensors for EV type identification and quantification. The QCM frequency shift caused by EVs is directly proportional to their concentration, while electroanalysis relies on measuring the curvature of the I−V curve as a distinguishing feature, which is also proportional to EV concentration. Linear regression lines for the QCM frequency shift and the electroanalysis curvature of various EV types are plotted separately, enabling the estimation of the corresponding concentration for an unknown EV type on the graphs. By intersecting the results from both biosensors, the unknown EV type can be identified. The biosensor analysis method proves to be an effective means of analyzing both the type and concentration of EVs from different cell lines.

## 1. Introduction

Unlike benign tumors, which typically remain localized and can often be surgically removed, malignant tumors involve abnormal cell proliferation that may infiltrate other organs in the body, thereby impacting the patient’s physiological functions [1]. Consequently, malignant tumors are also referred to as cancers. Currently, cancer has emerged as one of the most prevalent diseases, encompassing over 200 different types and affecting more than 60 human organs [2]. Current methods for cancer detection encompass various biochemical approaches, such as tomography [3,4], magnetic resonance imaging (MRI) [5,6], ultrasound [7,8], endoscopic examinations [9], and mass/optical spectroscopies [10,11]. However, these conventional methods often lack sufficient sensitivity and specificity and require expensive equipment. The timing of cancer diagnosis significantly impacts its treatment outcome. Studies have demonstrated that prolonging the diagnostic period from 31 to 151 days increases the mortality rate by 1.5 times, while delays exceeding 151 days elevate the mortality rate by 1.64 times [12]. The early detection of cancer metastasis could substantially enhance both cure rates and patient survival.

Recent studies have unveiled that both normal and pathological cells release extracellular vesicles (EVs), which are membrane-enclosed vesicles derived from cells, and that exosomes and ectosomes are two different types of EVs [13]. They play a crucial role in intercellular communication. Exosomes, lipid bilayer vesicles ranging from 50 to 100 nm in size, are released in the exocytosis of multivesicular bodies (MVBs) and carry extensive identity information about their cells of origin. Due to their ability to enter the body’s circulatory system, they are present in various biological fluids, including blood, urine, saliva, and breast milk [14]. Notably, exosomes released by cancer cells are particularly abundant in the blood of cancer patients. For instance, the vesicular material in patients with ovarian cancer increases by approximately 3–4 times [15]. Consequently, cancer-derived exosomes have gained widespread recognition as a novel category of diagnostic and prognostic biomarkers [16,17,18,19,20]. Various detection methods have been developed, including fluorescence assays [21,22,23], electrochemiluminescence [24], surface-enhanced Raman spectroscopy [25,26], and surface plasmon resonance [27]. On the other hand, ectosomes are vesicles with diameters ranging from 100 to 350 nm that bud directly from the plasma membrane and are shed into the extracellular space [28,29,30]. These vesicles are expelled outside the cell rather than within it. Ectosomes are believed to promote inflammation and cell death, and, in the context of cancer, they play roles in invasion and metastasis.

In this study, ectosomes extracted from A549 and MG63 cells were used as examples for analysis. A549 represents a human non-small-cell lung cancer cell line, and lung cancer stands as one of the most life-threatening diseases with persistently high mortality rates [31,32]. Due to numerous challenges in the early diagnosis of lung cancer, it is recognized as a major global public health concern. In 2018, deaths due to lung cancer constituted 18% of the total cancer-related deaths. The predominant causes attributed to lung cancer include smoking, environmental pollution, occupational exposure, etc. Studies indicate that 90–95% of lung cancers originate from epithelial cells in the bronchi, hence the term bronchogenic carcinoma. Lung cancer tends to metastasize early, spreading to various organs within the body, notably the adrenal glands, liver, brain, and bones. Over the past two decades, the 5-year survival rate for lung cancer remains notably low, hovering around 15%. On the other hand, a bone tumor occurs when cells within the bone divide uncontrollably and form abnormal tissue masses. Some bone tumors are malignant, often originating from cancers in other parts of the body, such as lung, breast, thyroid, kidney, and prostate cancer, and they may metastasize or cause cancer cells to spread throughout the body. Osteoblast-like MG63 is a human osteosarcoma cell line [33,34].

Two methods to identify and quantify ectosomes were proposed and compared in this study. The first approach proposed for ectosome identification involves the use of sodium dodecyl sulfate–polyacrylamide gel electrophoresis (SDS-PAGE). EVs bear numerous proteins on their surface, and these proteins exhibit distinct expression profiles in EVs secreted by various types of cancer cells [35]. Several studies have highlighted their potential as biomarkers for cancer detection. For instance, research conducted by Sandfeld-Paulsen [36] identified proteins such as CD151 and CD171 as viable biomarkers capable of distinguishing between lung cancer and non-lung cancer patients. Similarly, Koh’s study [37] identified CD63 as a prognostic marker for non-small-cell lung cancer (NSCLC), suggesting that low CD63 expression might serve as an adverse prognostic factor for NSCLC patients. Consequently, exosomal proteins not only confirm the presence of exosomes but also hold promise in identifying specific types of cancer cells [38]. Among these proteins, tetraspanins are noteworthy. Their content differs between normal cells and cancer cells, making the measurement of tetraspanins a crucial method for detecting both EVs and cancer.

The second method employed in this study involves two measurements of ectosomes using biosensors: quartz crystal microbalance (QCM) measurement and electroanalysis measurement. The QCM is a mass-sensitive device on which mass accumulation and/or changes can induce an oscillation frequency shift. Therefore, QCM serves as a sensitive method for detecting EV content [39,40]. On the other hand, electroanalytical methods are employed to measure the electrical parameters of the analyte solutions, which are indicative of either the identity or quantity of the analyte present, enabling the precise detection of EV content [41]. Through these two biosensor measurements, the type and content of ectosomes in different cell lines can be accurately identified.

The methods proposed in this study are applicable to the analysis of ectosomes released from various cell lines, not limited to the two specific cell lines mentioned. Moreover, these methods can be used for the analysis of exosomes. Despite differences in the origin sites and membrane sources between exosomes and ectosomes, these two categories of EVs exhibit similar functions upon release. Through biosensor analysis with QCM and IDT chips, it is possible to quickly identify and quantify ectosomes (or exosomes) from different cell lines.

## 2. Materials and Methods

### 2.1. Materials

For the SDS-PAGE analysis, ExoQuick-TC, a commercial kit from System Biosciences (SBI) (Palo Alto, CA, USA), was utilized to isolate exosomes, and Radio Immunoprecipitation Assay (RIPA) lysis buffer purchased from Sigma-Aldrich (Burlington, MA, USA) was employed to lyse cells and tissue.

Reagents for the biofunctionalization process of the QCM included 11-mercaptoundecanoic acid (MUA) purchased from Sigma-Aldrich, 1-(3-Dimethylaminopropyl)-3-ethylcarbodiimide hydrochloride (EDC) obtained from Alfa Aesar (Ward Hill, MA, USA), N-Hydroxysuccinimide (NHS) purchased from Acros Organics (Geel, Belgium), and bovine serum albumin (BSA) purchased from Medicago (Quebec City, QC, Canada). Moreover, reagents for the biofunctionalization process of the electroanalysis device included (3-Aminopropyl)trimethoxysilane (APTES) and Glutaraldehyde (GA), both purchased from Alfa Aesar. Additionally, polydimethylsiloxane (PDMS, SYLGARD^®^184), purchased from Dow Corning (Midland, MI, USA), was used for the fabrication of the transducer.

### 2.2. Method 1: SDS-PAGE Analysis

To assess tetraspanins on EVs’ surfaces, supernatants were separately collected from A549 and MG63 cell cultures. Various methods exist for purifying EVs, and in this study, ExoQuick-TC was employed to isolate EVs. This kit induces EV precipitation due to the presence of a super-hydrophilic polymer solution, reducing EV solubility. Hence, EV isolation was achieved using low-speed centrifugation.

Next, the proteins CD63 and CD81 present on the EVs were extracted using RIPA lysis buffer, which enables rapid, efficient cell lysis and solubilization of proteins from both adherent and suspension-cultured mammalian cells. Subsequently, they underwent sodium dodecyl sulfate polyacrylamide gel electrophoresis (SDS-PAGE) analysis.

### 2.3. Method 2: Biosensor Measurements

#### 2.3.1. Measurements by QCM

In this study, a commercially available crystal oscillator with a 10 MHz miniature AT-cut single quartz crystal sandwiched between two silver-plated electrodes enclosed in a hermetical seal was used as the QCM substrate. Upon removing the hermetical metal seal and thoroughly cleaning the QCM, a self-assembling monolayer (SAM) of thiol derivative was formed on the QCM surface using 20 mM MUA. Subsequently, 20 μL of the MUA solution was dropped onto the QCM electrode and heated in a 37 °C oven for 30 min. Following rinsing with deionized (DI) water, the QCM was immersed in a solution containing 0.8 M EDC and 0.2 M NHS in ambient light at room temperature for 1 h to activate the thiol (-SH) layer of the SAM. After another rinse with deionized water, the QCM underwent dehydration baking at 37 °C to dry the QCM surface. The final step before the bioassay involved immobilizing CD81 antibodies on the QCM. Initially, 10 µL of CD81 antibody stock solution (100 µg/200 µL) was diluted to 5 µg/250 µL using 10 mM PBST. Subsequently, 15 µL of the solution was dropped onto the QCM electrode and heated in a 37 °C oven for 1 h. Following the immobilization step, the QCM was rinsed with PBST and deionized water to remove any residual but unbound antibodies. To prevent nonspecific adsorption on the QCM surface, the blocking step involved spraying a 0.4% BSA solution onto the QCM electrode at room temperature. At this point, the QCM was ready for bioassays.

#### 2.3.2. Measurements by Electroanalysis

**Figure 1:**
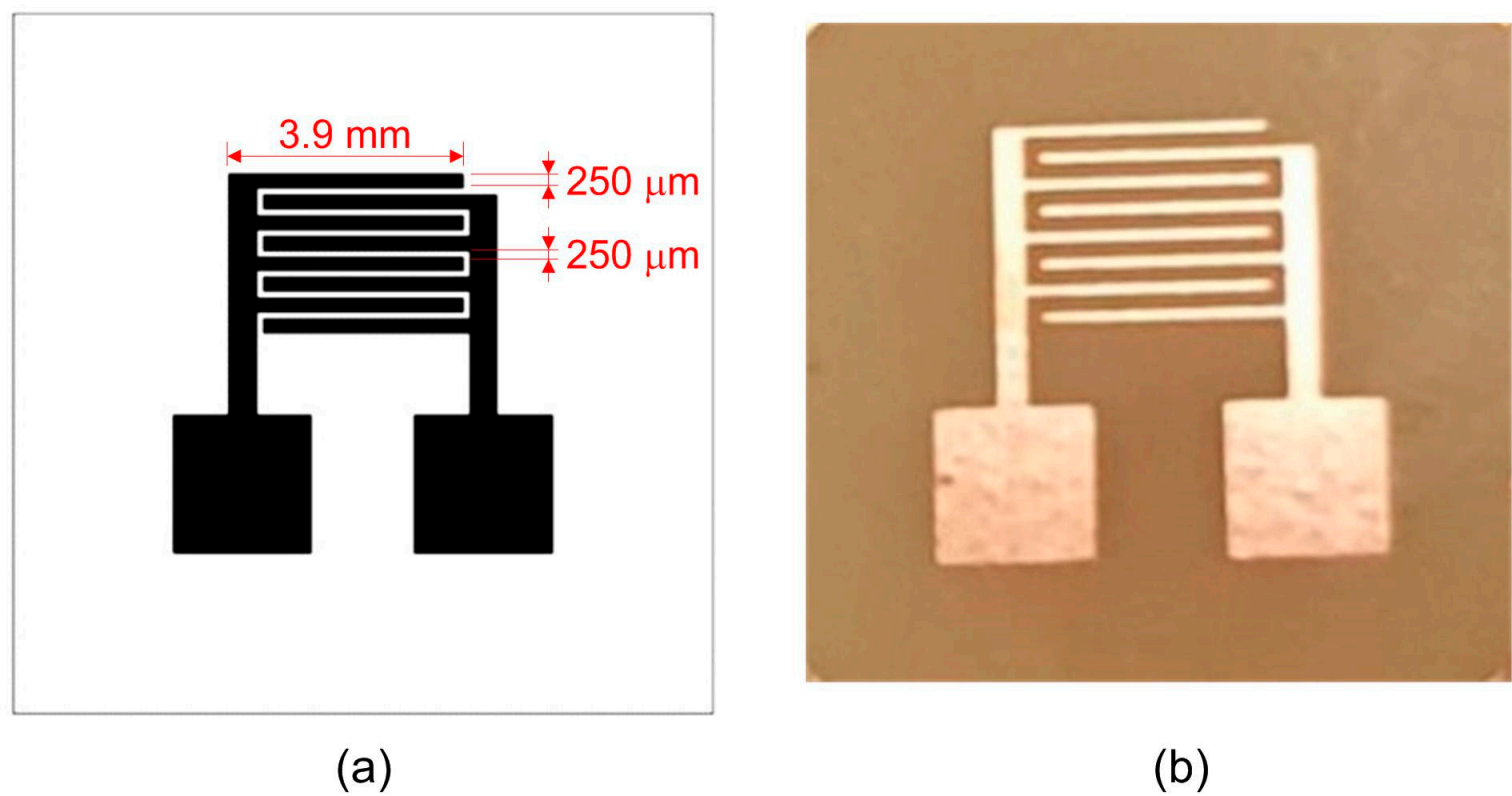
Interdigital transducer (IDT) for the electroanalysis measurement: (**a**) IDT dimensions. (**b**) Photo of IDT chip.

Electroanalysis of the supernatant was conducted using a biosensor comprising an interdigital transducer (IDT), a PDMS layer, and ZnO nanowires. The IDT device, composed of two interlocking comb-shaped arrays of electrodes, featured 4 interdigitated fingers on each side, with a finger length of 3.9 mm, a width of 250 μm, and a gap of 250 μm, as shown in Figure 1. Fabrication of this IDT structure involved etching the copper foil of a printed circuit board (PCB) through conventional PCB processing procedures. Subsequently, a PDMS layer with a punched hole was applied to cover the IDT, exposing the comb-shaped electrodes for the synthesis of ZnO nanowires. The punched hole within this PDMS layer also served to confine the region for the bioassay.

The synthesis of ZnO nanowires aimed to enhance measurement sensitivity by utilizing free-standing ZnO nanowires extending upward, thereby offering an increased surface area for antibody immobilization to facilitate EV detection. To achieve this, crystal growth of ZnO nanowires was conducted inside the punched hole using the conventional hydrothermal method.

The biofunctionalization process aimed to immobilize antibodies onto the surface of ZnO nanowires to facilitate bonding with the antigens present on EVs through antigen–antibody interactions. The initial step of biofunctionalization involved applying a solution of pure ethanol containing 4% APTES onto the IDT region. Subsequently, the biosensor was placed in a 70 °C hot circulator oven for 1 h, followed by rinsing with pure ethanol to remove any unbound APTES molecules. Following this, a solution of 2.5% GA in DI water was applied to the same IDT area and allowed to react for 1 h at room temperature. The biosensor was then rinsed with DI water to eliminate any unbound GA molecules. Subsequent to this modification step, 15 μL of antibody solution (20 μg/mL anti-CD81 in 10 mM PBS) was dropped onto the IDT. The biosensor underwent an incubation period in a hot circulator oven at 37 °C for 2.5 h to enable the covalent bonding of the anti-CD81 to the nanowire surface. The remaining anti-CD81 residues were washed out using a washing buffer (PBST). Finally, to prevent nonspecific protein binding, a blocking step was implemented by applying 15 μL of 1% BSA for 1 h. Subsequently, any residual BSA was washed away using PBST.

## 3. Results and Discussion

### 3.1. Analysis by NTA and ELISA Kit

**Figure 2:**
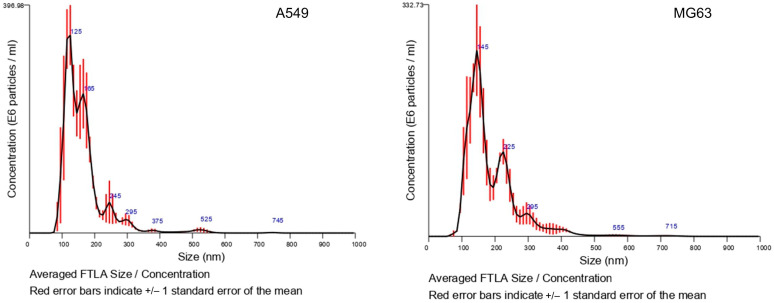
The size distribution and concentration of nanoparticles in A549 and MG63 supernatants measured using NanoSight LM10 instrument (The red lines represent the measurement errors).

Prior to the experiments, a NanoSight LM10 instrument (Malvern Instruments Ltd., Malvern, UK), utilizing Nanoparticle Tracking Analysis (NTA) technology, was used to determine the size distribution and concentration of various nanoparticles isolated by ExoQuick-TC. The volume of supernatant used was 0.3 mL. As shown in Figure 2, the size of the isolates from both A549 and MG63 cells was greater than 100 nm, with an average size of 159.6 nm for A549 and 185.0 nm for MG63, indicating that the isolates were all classified as ectosomes. Moreover, the estimated concentration of EVs from A549 cells via NTA was denoted as $2.86\times 10^{9}$ particles/mL, while that from MG63 cells was denoted as $2.54\times 10^{9}$ particles/mL. The size distribution and concentration of EVs measured from different culture batches will exhibit slight variations. Therefore, measuring the particle count per milliliter alone is insufficient to distinguish between the two; further qualitative tests are required to identify the type of cells from which the EVs are released.

**Figure 3:**
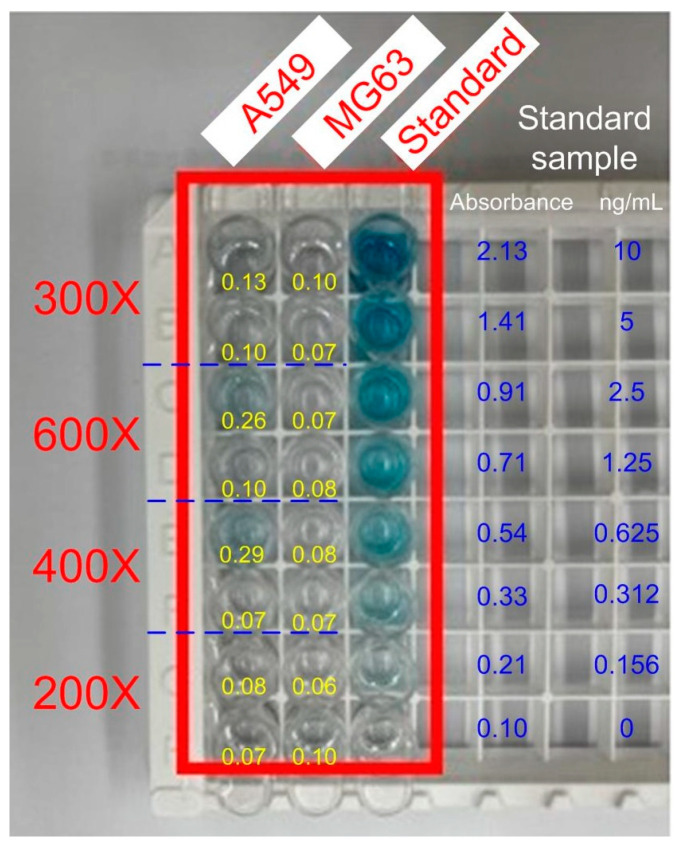
Measurement of CD63 using commercial ELISA kit (CSB-EL004960HU).

Furthermore, a commercially available ELISA kit (CSB-EL004960HU, purchased from Cusabio (Houston, TX, USA)) was employed to measure CD63. According to the protocol of the Cusabio ELISA kit, a 2-fold dilution series was prepared using the standard solution, and the corresponding absorbance values were obtained as shown in Figure 3. In addition, the supernatants from A549 and MG63 were individually diluted at ratios of 200, 300, 400, and 600. After lysing the cells with RIPA buffer, 100 µL of each diluted sample was added to the well of the ELISA kit. At different dilution ratios, both A549 and MG63 exhibited almost no coloration or minimal color development. In Figure 3, the yellow values represent the absorbance of each diluted sample. When compared to the absorbance values of standard samples, the corresponding concentrations for both A549 and MG63 were nearly zero, indicating undetectability. Evidently, for supernatants with even lower concentrations, measurement becomes more challenging and is practically unfeasible.

Therefore, both of the above two methods are ineffective in quantifying and distinguishing different EVs.

### 3.2. Method 1: SDS-PAGE Analysis

**Figure 4:**
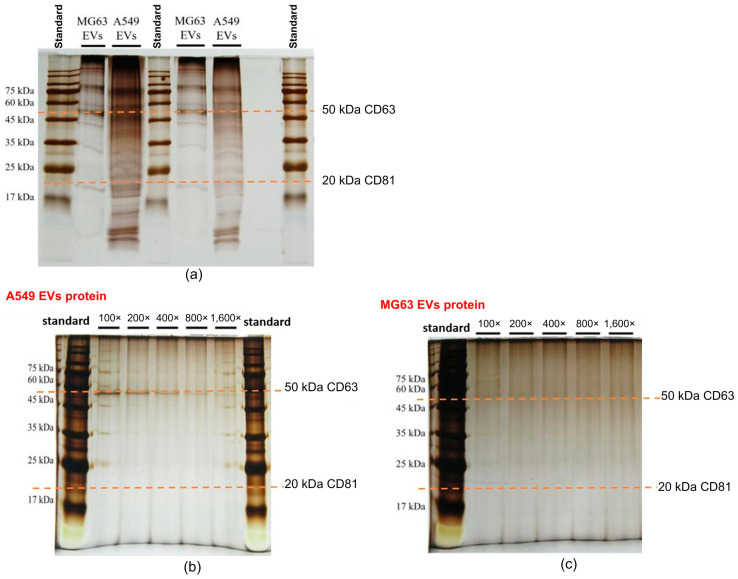
SDS-PAGE analysis: (**a**) Compared with the standard liquid, the molecular mass of CD63 is 50 kDa, while that of CD81 falls within the range of 20–25 kDa. (**b**) Electrophoresis results of A549 EV proteins at different dilution factors. (**c**) Electrophoresis results of MG63 EV proteins at different dilution factors.

SDS-PAGE analysis was employed to differentiate types of EVs by separating the molecular masses of the tetraspanins on the EVs’ surfaces. The results of the SDS-PAGE analysis are shown in Figure 4a. In this analysis, a standard solution served as a reference for different molecular masses, where CD63 exhibited a molecular mass of 50 kDa and CD81 displayed a molecular mass within the range of 20–25 kDa.

The proteins extracted from EVs of A549 and MG63 cells underwent electrophoresis at different dilutions (100-fold, 200-fold, 400-fold, 800-fold, and 1600-fold), and the outcomes are shown in Figure 4b,c. Grayscale values were then acquired using computer image processing.

**Figure 5:**
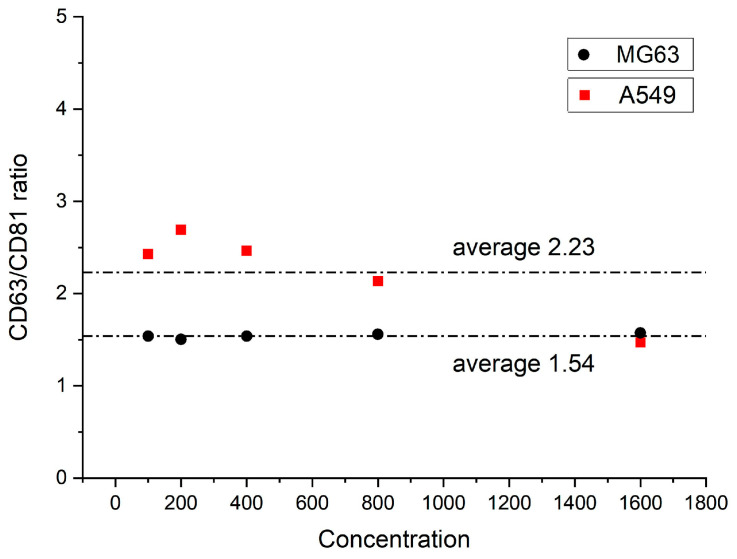
The ratio of CD63 to CD81 remains constant for EVs of the same type under various dilution factors, while variations are observed among different EV populations.

In Figure 5, pertaining to the same category of EVs, it is demonstrated that the grayscale values for the ratio of CD63 to CD81 at different dilutions exhibit approximate similarity, with an average value of about 2.23 for A549 and 1.54 for MG63. Remarkably, significant variations exist among distinct EVs. This experimental data indicates that the presence of CD63 and CD81 on a specific EV is in a consistent proportion. Nevertheless, the quantity of CD63 and CD81 on EVs secreted by different cancer cells varies considerably. Therefore, the ratio of CD63 to CD81 can serve as a distinguishing characteristic for cancer cell-secreted EVs, aiding in the differentiation between types of cancer cells.

However, it is crucial to acknowledge that in image processing, the positional selection is visually conducted. Consequently, variations occurring during electrophoresis and imaging processes might introduce a certain degree of error into the concentration values. Furthermore, this method lacks effectiveness in measuring low concentrations. Nevertheless, it does offer reference values for extracting characteristics from various types of cancer cells in subsequent analyses (not confined solely to CD63 and CD81).

### 3.3. Method 2: Biosensor Measurements

#### 3.3.1. Measurements by QCM

**Figure 6:**
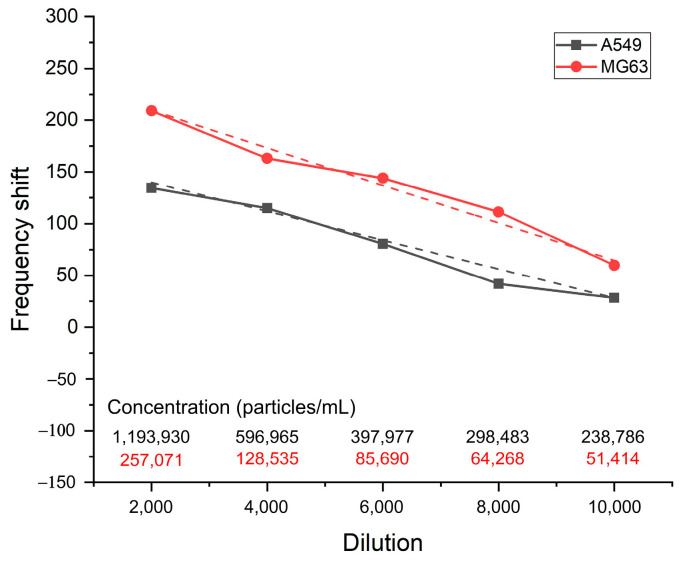
The frequency shift of QCM is inversely proportional to the dilution factor of EVs (a larger dilution ratio corresponds to a lower concentration).

After diluting the supernatant by factors of 2000, 4000, 6000, 8000, and 10,000, the frequency shift induced by adding the supernatant was measured using the Agilent 53,200 frequency counter (Agilent Technologies, Inc., Santa Clara, CA, USA). The experimental results indicate a direct relationship between the concentration of the EVs and the magnitude of the frequency shift: higher EV concentrations correspond to greater frequency shifts, as shown in Figure 6. Furthermore, from Figure 6, it can be observed that the concentration of A549, diluted 10,000 times (238,786 particles/mL), is approximately equal to the concentration of MG63, diluted 2000 times (257,071 particles/mL). However, A549 has a frequency shift of 28.4333 Hz, while MG63 has a frequency shift of 209 Hz. Different EVs exhibit distinct frequency shifts, and each type of EV has its own linear regression equation, as follows:

(1) A549: y=167.7853−0.01395x

(2) MG63: y=245.58128−0.0181x

where y is the frequency shift (Hz) induced by EVs and x is the dilution factor.

#### 3.3.2. Measurements by Electroanalysis

To investigate the electrical properties of EVs across various concentrations, electrical measurements were conducted using a PE-4 probe station in conjunction with a Keithley 2614B SourceMeter. The current–voltage (I–V) relationship was assessed by employing a two-probe system. In this setup, a controlled voltage was applied to the source electrode of the IDT device, while the drain electrode was grounded as a reference. The applied voltage spanned from −1 V to 0 V, incremented in 0.02 V intervals, to characterize the I–V relationship.

**Figure 7:**
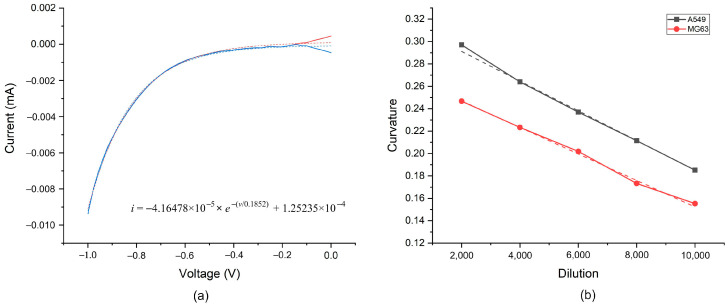
Electroanalysis measurements: (**a**) The I–V curves were measured by applying a voltage range from −1 V to 0 V, with increments of 0.02 V. (**b**) The curvature parameter was inversely proportional to the dilution ratio of EVs.

The I–V curve of the diluted A549 supernatant, measured at a 2000-fold dilution, is shown in Figure 7a. The dashed line represents the curve fitted using an exponential function $i=i_{0}+Ae^{-\frac{v}{\tau }}$, wherein v is the applied voltage. Due to the extremely small values of $i_{0}$ and A in the fitting curve, on the order of 10^−5^~10^−7^, and as the parameter τ affects the curvature of the curve, we chose τ as the sole representative factor for electrical measurements. Experimental results indicate that with different dilution factors of the supernatant, as the dilution factor increases (indicating lower extracellular vesicle concentrations), the τ value decreases. This exhibits a linear positive correlation between the dilution factor and τ, as shown in Figure 7b. The linear regression equations are as follows.

(3) $$A549\text{:}\;\tau =0.31771-(1.32673\times 10^{-5})x$$

(4) $$\mathrm{MG}63\text{:}\;\tau =0.27062-(1.18332\times 10^{-5})x$$

where τ is the curvature and x is the dilution factor.

#### 3.3.3. Identification of EVs

**Scheme 1:**
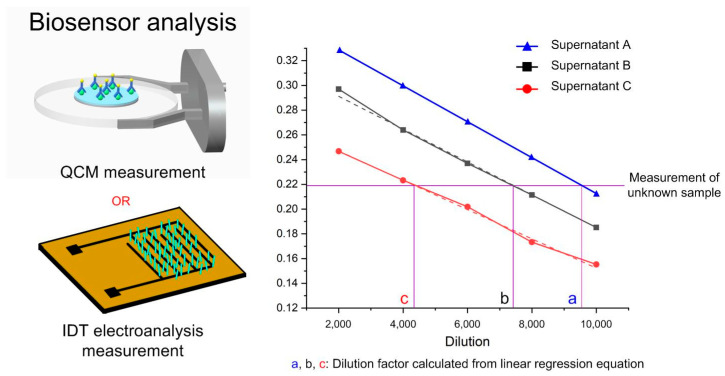
The measurement of frequency shift for QCM (or curvature for IDT) of an unknown cell line supernatant indicates that it is either supernatant A at dilution a, supernatant B at dilution b, or supernatant C at dilution c, etc.

Currently, there exist numerous methods for detecting types of extracellular vesicles. This study demonstrates the use of QCM and electroanalytical measurements to differentiate various types of extracellular vesicles efficiently, assuming that the linear relationships between the measurements (frequency shift for QCM or curvature for IDT) of EVs in the supernatants from various cell lines and their dilution factors (i.e., concentration) have been established, as shown in Scheme 1. For an unknown cell line supernatant with an unknown concentration measured using QCM or IDT, the intersection of the measurement result with these lines can indicate that it is either supernatant A at dilution a, supernatant B at dilution b, or supernatant C at dilution c, etc. Therefore, compared with another measurement result, the type and concentration of the supernatant of an unknown cell line can be inferred.

**Figure 8:**
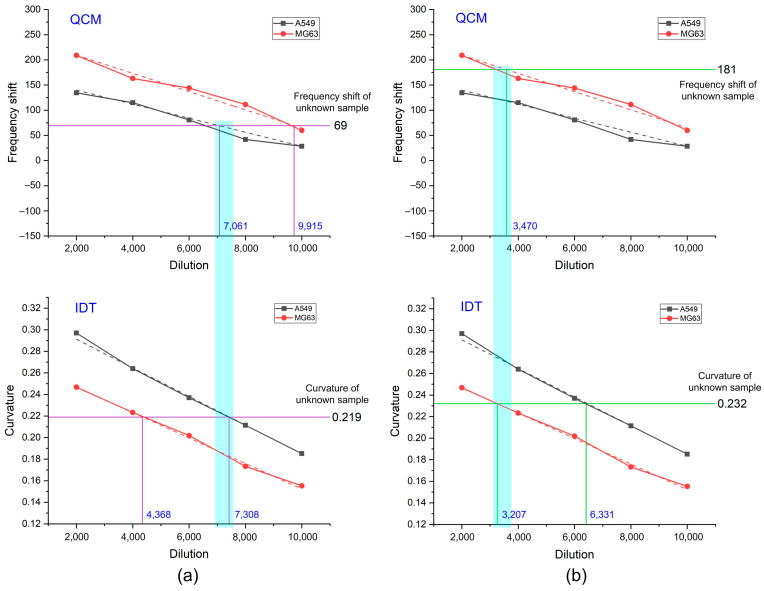
Identification of unknown EV sample using biosensor measurements: (**a**) QCM and IDT measurements infer that the unknown sample is more likely A549 supernatant, with an estimated average dilution factor of approximately 7185 times. (Experimental sample: 7000-fold dilution of A549 supernatant.) (**b**) Experimental sample: 3000-fold dilution of MG63 supernatant.

The experiment involved diluting the A549 supernatant 7000 times (treated as an unknown sample) for QCM and electrical measurements. The recorded frequency shift was 69, and the τ value was 0.2192867. As shown in Figure 8a, the analysis of the QCM frequency shift plot and the corresponding fitting equations suggested that the observed frequency shift in the unknown sample could potentially result from a 7061-fold dilution of the A549 supernatant or a 9915-fold dilution of the MG63 supernatant. Similarly, the electrical τ value indicated that the unknown sample might be the A549 supernatant diluted approximately 7308 times or the MG63 supernatant diluted around 4368 times. By comparing these results, it was inferred that the unknown sample was more likely the A549 supernatant, with an estimated average dilution factor of approximately 7185 times (corresponding to a concentration of approximately 332,340 particles/μL), consistent with the actual reagent used.

Upon diluting the MG63 supernatant 3000 times and treating it as an unknown sample, repeating the experiment resulted in a frequency shift of 181 and a τ value of 0.2326133. An analysis based on the plots and the fitting equations suggested that the observed frequency shift might arise from either the A549 supernatant at a dilution of less than 2000 times (estimated by the fitting equation as a 1594-fold dilution) or the MG63 supernatant at a dilution of 3470 times, as shown in Figure 8b. Additionally, the τ value possibly originated from a 6331-fold diluted A549 supernatant or an MG63 supernatant at a dilution of 3207 times. The experimental results strongly indicated that the unknown sample was more likely to be MG63 supernatant, with an estimated average dilution factor of approximately 3338 times (corresponding to a concentration of approximately 154,026 particles/μL), mirroring the actual reagent used.

Through the utilization of QCM and electroanalytical measurements, the EV type within the unknown sample can be identified by comparing the fitted lines for A549 and MG63. Additionally, an estimation of the concentration of the unknown sample becomes feasible. Despite this experiment’s focus on two specific types of supernatants, the establishment of fitted curves for various extracellular vesicles enables the potential identification of corresponding extracellular vesicles among a broader range using QCM and electroanalytical measurements.

## 4. Conclusions

This study proposes and compares two methods for identifying the types and concentrations of EVs in different cell lines, yielding two key findings. Through SDS-PAGE analysis, a constant and distinct ratio of CD63 to CD81 was observed in different EVs, about 2.23 for A549 and 1.54 for MG63. It can serve as a significant characteristic of EVs secreted by cancer cells. However, the electrophoresis and imaging processes may introduce some degree of error in the concentration values, particularly at lower concentrations, potentially limiting the effectiveness of this method. Another discovery is that the QCM frequency shift caused by EVs and the curvature of the I−V curve measured during electroanalysis are both directly proportional to the EV concentration. Both QCM and IDT electroanalysis measurements exhibit excellent sensitivity even at low concentrations of EVs. Therefore, this biosensor technique with QCM and IDT chips proves effective in analyzing the types and concentrations of EVs from different cell lines. By establishing a database of EVs released by different cells of interest using this biosensor technique, it is possible to quickly identify the cell type of unknown EVs. While this study specifically analyzed ectosomes extracted from A549 and MG63 cells, the proposed biosensor method is applicable to the analyses of ectosomes from other cell types and is not limited to these two specific cell lines. This method can also be used for the analyses of exosomes.

## Data Availability

The data presented in this study are available upon request from the corresponding author.
